# Supplementary material for: Resolving the full spectrum of human genome variation using Linked-Reads
Source: Genome Res. 2019 Apr;29(4):635–45. doi: 10.1101/gr.234443.118 (PMC6442396; doi:10.1101/gr.234443.118)
Supplement: Supplemental Material [file supp_gr.234443.118_Supplemental_longranger-2.2.zip › longranger-2.2.tar.bz2!/lr2.2-public/lib/stan/cmdstan/stan/doc/api/html/classstan_1_1mcmc_1_1chains-members.html]

Stan: Member List


|  |  |
| --- | --- |
|  | Stan  2.5.0  probability, sampling & optimization |


- Main Page
- Related Pages
- Namespaces
- Classes
- Files

- Class List
- Class Index
- Class Hierarchy
- Class Members

All Classes Namespaces Files Functions Variables Typedefs Enumerations Enumerator Friends Macros Pages

- stan
- mcmc
- chains

stan::mcmc::chains< RNG > Member List

This is the complete list of members for stan::mcmc::chains< RNG >, including all inherited members.

|  |  |  |
| --- | --- | --- |
| add(const int chain, const Eigen::MatrixXd &sample) | stan::mcmc::chains< RNG > | inline |
| add(const Eigen::MatrixXd &sample) | stan::mcmc::chains< RNG > | inline |
| add(const std::vector< std::vector< double > > &sample) | stan::mcmc::chains< RNG > | inline |
| add(const stan::io::stan\_csv &stan\_csv) | stan::mcmc::chains< RNG > | inline |
| autocorrelation(const int chain, const int index) const | stan::mcmc::chains< RNG > | inline |
| autocorrelation(int chain, const std::string &name) const | stan::mcmc::chains< RNG > | inline |
| autocovariance(const int chain, const int index) const | stan::mcmc::chains< RNG > | inline |
| autocovariance(int chain, const std::string &name) const | stan::mcmc::chains< RNG > | inline |
| central\_interval(int chain, int index, double prob) const | stan::mcmc::chains< RNG > | inline |
| central\_interval(int index, double prob) const | stan::mcmc::chains< RNG > | inline |
| central\_interval(int chain, const std::string &name, double prob) const | stan::mcmc::chains< RNG > | inline |
| central\_interval(const std::string &name, double prob) const | stan::mcmc::chains< RNG > | inline |
| chains(const Eigen::Matrix< std::string, Eigen::Dynamic, 1 > &param\_names) | stan::mcmc::chains< RNG > | inline |
| chains(const std::vector< std::string > &param\_names) | stan::mcmc::chains< RNG > | inline |
| chains(const stan::io::stan\_csv &stan\_csv) | stan::mcmc::chains< RNG > | inline |
| correlation(const int chain, const int index1, const int index2) const | stan::mcmc::chains< RNG > | inline |
| correlation(const int index1, const int index2) const | stan::mcmc::chains< RNG > | inline |
| correlation(const int chain, const std::string &name1, const std::string &name2) const | stan::mcmc::chains< RNG > | inline |
| correlation(const std::string &name1, const std::string &name2) const | stan::mcmc::chains< RNG > | inline |
| covariance(const int chain, const int index1, const int index2) const | stan::mcmc::chains< RNG > | inline |
| covariance(const int index1, const int index2) const | stan::mcmc::chains< RNG > | inline |
| covariance(const int chain, const std::string &name1, const std::string &name2) const | stan::mcmc::chains< RNG > | inline |
| covariance(const std::string &name1, const std::string &name2) const | stan::mcmc::chains< RNG > | inline |
| effective\_sample\_size(const int index) const | stan::mcmc::chains< RNG > | inline |
| effective\_sample\_size(const std::string &name) const | stan::mcmc::chains< RNG > | inline |
| index(const std::string &name) const | stan::mcmc::chains< RNG > | inline |
| mean(const int chain, const int index) const | stan::mcmc::chains< RNG > | inline |
| mean(const int index) const | stan::mcmc::chains< RNG > | inline |
| mean(const int chain, const std::string &name) const | stan::mcmc::chains< RNG > | inline |
| mean(const std::string &name) const | stan::mcmc::chains< RNG > | inline |
| num\_chains() const | stan::mcmc::chains< RNG > | inline |
| num\_kept\_samples(const int chain) const | stan::mcmc::chains< RNG > | inline |
| num\_kept\_samples() const | stan::mcmc::chains< RNG > | inline |
| num\_params() const | stan::mcmc::chains< RNG > | inline |
| num\_samples(const int chain) const | stan::mcmc::chains< RNG > | inline |
| num\_samples() const | stan::mcmc::chains< RNG > | inline |
| param\_name(int j) const | stan::mcmc::chains< RNG > | inline |
| param\_names() const | stan::mcmc::chains< RNG > | inline |
| quantile(const int chain, const int index, const double prob) const | stan::mcmc::chains< RNG > | inline |
| quantile(const int index, const double prob) const | stan::mcmc::chains< RNG > | inline |
| quantile(int chain, const std::string &name, double prob) const | stan::mcmc::chains< RNG > | inline |
| quantile(const std::string &name, const double prob) const | stan::mcmc::chains< RNG > | inline |
| quantiles(int chain, int index, const Eigen::VectorXd &probs) const | stan::mcmc::chains< RNG > | inline |
| quantiles(int index, const Eigen::VectorXd &probs) const | stan::mcmc::chains< RNG > | inline |
| quantiles(int chain, const std::string &name, const Eigen::VectorXd &probs) const | stan::mcmc::chains< RNG > | inline |
| quantiles(const std::string &name, const Eigen::VectorXd &probs) const | stan::mcmc::chains< RNG > | inline |
| samples(const int chain, const int index) const | stan::mcmc::chains< RNG > | inline |
| samples(const int index) const | stan::mcmc::chains< RNG > | inline |
| samples(const int chain, const std::string &name) const | stan::mcmc::chains< RNG > | inline |
| samples(const std::string &name) const | stan::mcmc::chains< RNG > | inline |
| sd(const int chain, const int index) const | stan::mcmc::chains< RNG > | inline |
| sd(const int index) const | stan::mcmc::chains< RNG > | inline |
| sd(const int chain, const std::string &name) const | stan::mcmc::chains< RNG > | inline |
| sd(const std::string &name) const | stan::mcmc::chains< RNG > | inline |
| set\_warmup(const int chain, const int warmup) | stan::mcmc::chains< RNG > | inline |
| set\_warmup(const int warmup) | stan::mcmc::chains< RNG > | inline |
| split\_potential\_scale\_reduction(const int index) const | stan::mcmc::chains< RNG > | inline |
| split\_potential\_scale\_reduction(const std::string &name) const | stan::mcmc::chains< RNG > | inline |
| variance(const int chain, const int index) const | stan::mcmc::chains< RNG > | inline |
| variance(const int index) const | stan::mcmc::chains< RNG > | inline |
| variance(const int chain, const std::string &name) const | stan::mcmc::chains< RNG > | inline |
| variance(const std::string &name) const | stan::mcmc::chains< RNG > | inline |
| warmup() const | stan::mcmc::chains< RNG > | inline |
| warmup(const int chain) const | stan::mcmc::chains< RNG > | inline |


---

[ Stan Home Page ]

*© 2011–2014,
Stan Development Team.*
